# Integrated Analysis of lncRNA–Mediated ceRNA Network in Lung Adenocarcinoma

**DOI:** 10.3389/fonc.2020.554759

**Published:** 2020-09-15

**Authors:** Xianxian Wu, Zhilin Sui, Hongdian Zhang, Ying Wang, Zhentao Yu

**Affiliations:** Department of Esophageal Cancer, Tianjin Medical University Cancer Institute and Hospital, National Clinical Research Center for Cancer, Key Laboratory of Cancer Prevention and Therapy, Tianjin’s Clinical Research Center for Cancer, Tianjin, China

**Keywords:** bioinformatics, lung adenocarcinoma, competitive endogenous RNA, long non-coding RNAs, prognosis

## Abstract

**Background:**

A growing body of evidence indicates that long non-coding RNAs (lncRNAs) can act as competitive endogenous RNAs (ceRNAs) to bind to microRNAs (miRNAs), thereby affecting and regulating the expression of target genes. The lncRNA–miRNA–mRNA ceRNA network has been theorized to play an indispensable role in many types of tumors. However, the role of the lncRNA-related ceRNA regulatory network in lung adenocarcinoma (LUAD) remains unclear.

**Methods:**

We downloaded the RNAseq and miRNAseq data of LUAD from The Cancer Genome Atlas (TCGA) data portal and identified differentially expressed lncRNAs (DElncRNAs), differentially expressed miRNAs (DEmiRNAs), and differentially expressed mRNAs (DEmRNAs) between LUAD and corresponding paracancerous tissues by using the edgeR package of R software. We constructed the lncRNA–miRNA–mRNA ceRNA network by using Cytoscape (version 3.7.2) on the basis of the interaction generated from the miRcode, miRTarBase, miRDB, and TargetScan databases. Gene Ontology (GO) annotation and Kyoto Encyclopedia of Genes and Genomes (KEGG) pathway analysis were performed with DAVID 6.8 bioinformatics resources and plotted by using the ggplot2 package in R. The effect of genes on LUAD prognosis was assessed by applying the survival package in R in accordance with the Kaplan–Meier curve.

**Results:**

In total, 1645 DElncRNAs, 117 DEmiRNAs, and 2729 DEmRNAs were identified in LUAD. The LUAD-specific ceRNA network was composed of 157 nodes and 378 edges (329 DElncRNA–DEmiRNA interactions and 49 DEmiRNA–DEmRNA interactions). GO and KEGG pathway annotations suggested that the LUAD-specific ceRNA network was related to tumor-related molecular functions and pathways. Seven lncRNAs (DISC1-IT1, SYNPR-AS1, H19, LINC00460, LINC00518, DSCR10, and STEAP2-AS1), one miRNA (hsa-mir-31), and 16 mRNAs (ATAD2, OSCAR, KIF23, E2F7, PFKP, MCM4, CEP55, CBX2, CCNE1, CLSPN, CCNB1, CDC25A, EZH2, CHEK1, SLC7A11, and PBK) were revealed to be significantly correlated with overall survival.

**Conclusion:**

In this study, we described the potential regulatory mechanism of the progression of LUAD. We proposed a new lncRNA–miRNA–mRNA ceRNA network that could help further explore the molecular mechanisms of LUAD.

## Introduction

Lung cancer is one of the most frequent cancers worldwide (1). It is classified into small cell lung carcinoma (accounting for 15% of cases) and non-small cell lung carcinoma (NSCLC, accounting for 85% of cases) (2). NSCLC is further divided into three types: lung adenocarcinoma (LUAD), squamous cell carcinoma, and large cell carcinoma; LUAD is the main histological subtype of lung cancer and accounts for 40% of all cases (3). Despite advances in diagnosis and treatment in recent years, the overall survival rate of patients with LUAD remains unsatisfactory. Statistics show that the average 5-year survival rate is less than 20% (4). The insufficient understanding of the biological mechanism of LUAD limits the improvement of therapeutic effects. Therefore, further elucidating tumor pathogenesis and finding new biomarkers to improve prognosis are urgent.

Non-coding RNAs (ncRNAs), which constitute more than 90% of the RNA transcripts of the human genome, lack protein coding potential but are considered the key regulators of normal cell function and many diseases, including cancer (5). ncRNAs include microRNAs (miRNAs, 21–24 base pairs) and long non-coding RNAs (lncRNAs, longer than 200 base pairs) (6–9). As endogenous gene expression inhibitors, miRNAs can bind to the 3′ untranslated region of target RNAs to promote mRNA degradation or inhibit protein translation (10, 11). At present, miRNAs are widely accepted as oncogenes or tumor-suppressor genes that promote or inhibit the occurrence of tumors.

Long non-coding RNAs have been proven to be capable of regulating gene expression at multiple levels, namely, epigenetic, transcriptional, and post-transcriptional (12), and they are an indispensable participant in the development of cancer (13). In 2011, Salmena et al. (14) first proposed the competitive endogenous RNAs (ceRNA) hypothesis, which states that some RNAs, as ceRNAs, can regulate the expression of downstream mRNA by combining shared miRNAs. This hypothesis describes that ceRNAs can transform the function of target miRNAs by competing for the common binding sites of mRNAs on the target miRNAs. An increasing number of studies have shown that lncRNAs can act as ceRNAs to regulate target mRNA expression by competing for shared miRNAs. The schematic diagram of lncRNA-mediated ceRNA regulatory network is shown in Supplementary Figure 1. Several dysregulated expressed lncRNAs play an indispensable role in the development of tumors, showing their potential role in carcinogenesis and tumor inhibition (15, 16). An increasing amount of research reported that lncRNAs, as ceRNAs, are involved in the development of a variety of tumors, such as lymphoma (17), colorectal cancer (18), and gastric cancer (19). However, to date, research on the role of lncRNA-related ceRNA regulator networks in LUAD is rare.

In our study, the genome-wide expression profiles of lncRNAs, miRNAs, and mRNAs in patients with LUAD were screened through The Cancer Genome Atlas (TCGA) data portal. In addition, a LUAD-specific ceRNA network was established through comprehensive analysis. This network will help find new therapeutic targets and pathways for the treatment of patients and prolong the survival time of patients. Finally, we analyzed prognostic RNAs in the ceRNA network and found a biomarker that could be used to predict the survival of patients with LUAD.

## Materials and Methods

### Data Collection and Preprocessing

RNAseq data, miRNAseq data, and corresponding LUAD clinical data were downloaded from the TCGA data portal^1^. A total of 485 LUAD samples and 51 corresponding paracancerous tissue samples were collected. The following cases were excluded: (1) patients with other malignant tumors; (2) patients who had received preoperative treatment, such as chemotherapy; and (3) patients without complete clinical data. As a result, 251 LUAD samples and 19 paracancerous tissue samples were included in our study. Table 1 shows the clinicopathological data of 251 patients with LUAD.

**TABLE 1:** Clinicopathological characteristics of 251 LUAD patients.

Clinicopathological characteristics	Patients (*N* = 251)	
	*N*	%
**Age**		
<68	124	49.4
≥68	127	50.6
**Gender**		
Male	132	52.6
Female	119	47.4
**Pathologic stage**		
Stage I	129	51.4
Stage II	62	24.7
Stage III	44	17.5
Stage IV	16	6.4
**Pathologic T**		
T1	74	29.5
T2	139	55.4
T3	24	9.6
T4	14	5.6
**Pathologic N**		
N0	157	62.5
N1	54	21.5
N2	39	15.5
N3	1	0.4
**Pathologic M**		
M0	253	93.6
M1	16	6.4

### DElncRNAs, DEmiRNAs, and DEmRNAs in LUAD

We identified mRNAs and lncRNAs by using the Ensembl database. Differentially expressed lncRNAs (DElncRNAs), differentially expressed miRNAs (DEmiRNAs), and differentially expressed mRNAs (DEmRNAs) between LUAD samples and corresponding paracancerous tissues were analyzed and normalized by using the edgeR package in R. FDR <1%, | logFC| > 2, and *P* < 0.05 were used as thresholds. We used gplots package in R to generate volcano plots and heatmaps.

### Construction of a ceRNA Regulatory Network

The miRcode database^2^ was used to match DElncRNAs and DEmiRNAs. MiRNA target genes were predicted on the basis of three databases: miRTarBase^3^, miRDB^4^, and TargetScan^5^. Subsequently, we integrated the interaction between DEmiRNAs and DElncRNAs or DEmRNAs to construct a ceRNA regulatory network. Cytoscape (version 3.7.2) was used to visualize the ceRNA network.

### Functional Enrichment and Protein–Protein Interaction Analysis

DAVID 6.8 Bioinformatics Resources was used for Gene Ontology (GO) and Kyoto Encyclopedia of Genes and Genomes (KEGG) pathway annotations. In addition, bubble charts were plotted by using the ggplot2 package of R software. *P* < 0.05 was considered a threshold. Protein–protein interaction (PPI) network was established with The STRING online search tool, with a combined score ≥0.4. Cytoscape v3.7.2 was used to visualized the PPI network.

### Survival Analysis

Survival analysis was performed to assess the prognostic value of differentially expressed RNAs in LUAD by using the survival package in R. *P* < 0.05 was regarded as statistically significant.

## Results

### DElncRNAs, DEmiRNAs, and DEmRNAs in LUAD

**FIGURE 1:**
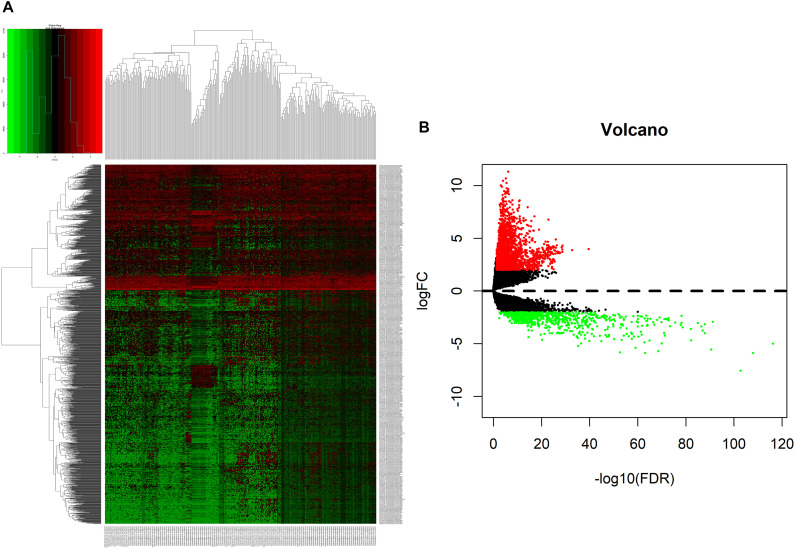
Identification of differentially expressed lncRNAs in LUAD and normal tissues. **(A)** The heatmap of genome-wide differentially expressed lncRNAs. **(B)** The volcano plot showed that a total of 1400 upregulated lncRNAs and 245 downregulated lncRNAs were screened out. Green and red represents downregulated and upregulated lncRNAs, respectively. lncRNAs, long non-coding RNAs; LUAD, lung adenocarcinoma.

**FIGURE 2:**
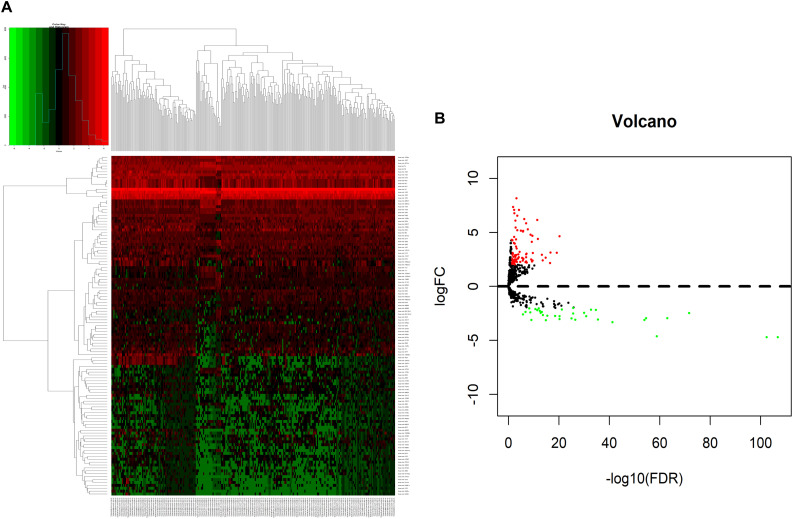
Identification of differentially expressed miRNAs in LUAD and normal tissues. **(A)** The heatmap of genome-wide differentially expressed miRNAs. **(B)** The volcano plot showed that a total of 82 upregulated miRNAs and 35 downregulated miRNAs were screened out. Green and red represents downregulated and upregulated miRNAs, respectively. miRNAs, microRNAs; LUAD, lung adenocarcinoma.

**FIGURE 3:**
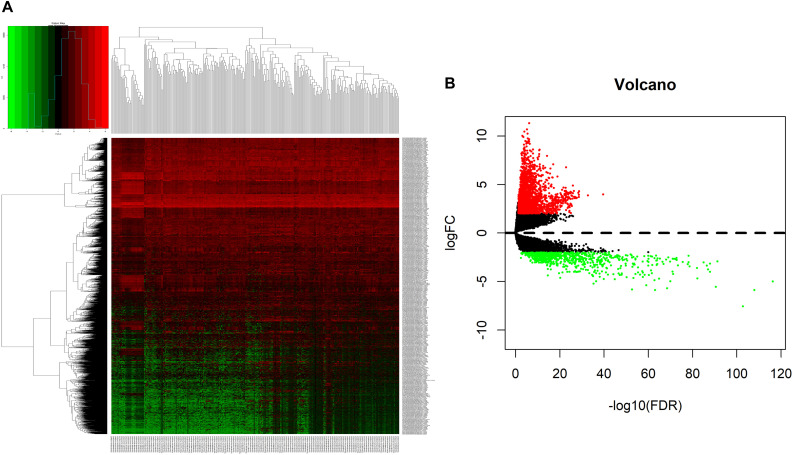
Identification of differentially expressed mRNAs in LUAD and normal tissues. **(A)** The heatmap of genome-wide differentially expressed mRNAs. **(B)** The volcano plot showed that a total of 2055 upregulated mRNAs and 674 downregulated mRNAs were screened out. Green and red represents downregulated and upregulated mRNAs, respectively. mRNAs, messenger RNAs; LUAD, lung adenocarcinoma.

We analyzed DElncRNAs, DEmiRNAs, and DEmRNAs between 251 LUAD samples and 19 corresponding paracancerous samples. A total of 1645 LUAD-specific lncRNAs (1400 upregulated and 245 downregulated, Figures 1A,B), 117 miRNAs (82 upregulated and 35 downregulated, Figures 2A,B), and 2729 mRNAs (2055 upregulated and 674 downregulated, Figures 3A,B) were identified as differentially expressed RNAs in LUAD. The top 10 DElncRNAs, DEmiRNAs, and DEmRNAs with their names, log2FC values, and *P*-values are listed in Supplementary Table 1.

### Construction of the ceRNA Network in LUAD

**FIGURE 4:**
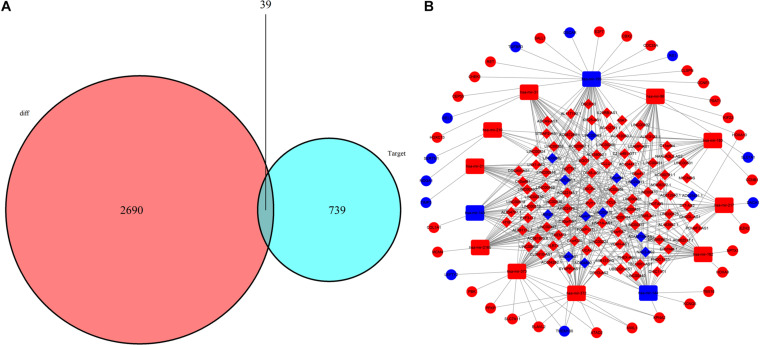
Construction of lncRNA-miRNA-mRNA network in LUAD. **(A)** Venn diagram of mRNAs included in the ceRNA network; the red area represents the number of DEmRNAs; the blue area shows the number of DEmRNAs targeted by DEmiRNAs. The purple area in the middle indicates the number of DEmRNAs included in ceRNA network. **(B)** lncRNA-miRNA-mRNA ceRNA network in LUAD. Red nodes indicate up-regulated RNAs while blue nodes indicate down-regulated RNAs. Diamond, round rectangle and ball represent DElncRNAs, DEmiRNAs, and DEmRNAs, respectively.

A total of 105 DElncRNAs and 13 DEmiRNAs were paired into 329 DElncRNA–DEmiRNA interactions, whereas 13 DEmiRNAs and 39 DEmRNAs were matched to form 49 pairs of DEmiRNA–DEmRNA interactions. Figure 4A shows the number of DEmRNA in the ceRNA network. Finally, the LUAD-specific lncRNA–miRNA–mRNA ceRNA regulatory network, which contained 157 nodes and 378 edges (Figure 4B), was constructed. Table 2 lists the top 10 DElncRNAs and their matching DEmiRNAs in the ceRNA network. All 105 DElncRNAs with their names, log2FC values, and FDR values are listed in Supplementary Table 2. The 13 DEmiRNAs with their matching DEmRNAs are presented in Table 3.

**TABLE 2:** The top 10 DElncRNAs in the ceRNA network were targeted by DEmiRNAs.

DElncRNAs	DEmiRNAs
DSCAM-AS1	hsa-mir-143
HOTAIR	hsa-mir-143, hsa-mir-21, hsa-mir-216b, hsa-mir-217
AC061975.6	hsa-mir-372, hsa-mir-373
CLDN10-AS1	hsa-mir-143
AC020907.1	hsa-mir-195, hsa-mir-216b
MIR137HG	hsa-mir-144, hsa-mir-182, hsa-mir-217, hsa-mir-31
LINC00392	hsa-mir-183
DSCR4	hsa-mir-144, hsa-mir-21, hsa-mir-216b, hsa-mir-31
LINC00501	hsa-mir-183
ERVMER61-1	hsa-mir-182, hsa-mir-21, hsa-mir-96

**TABLE 3:** The 13 DEmiRNAs with their target DEmRNAs in the ceRNA network.

DEmiRNAs	DEmRNAs
hsa-mir-372	MIXL1, ELAVL2, KPNA2, PFKP, SLC7A11, ATAD2, TMEM100
hsa-mir-96	SLC1A1
hsa-mir-144	KPNA2, HOXA10, KCNQ5, TBX18
hsa-mir-31	HOXC13, SELE
hsa-mir-195	HOXA10, CLSPN, RET, E2F7, RS1, CEP55, OSCAR, CCNE1, SALL1, PSAT1, KIF23, CHEK1, TGFBR3, CBX2, TMEM100, CDC25A
hsa-mir-210	SERTM1, MDGA1
hsa-mir-217	EZH2, DACH1
hsa-mir-21	TIMP3
hsa-mir-182	NPTX1, HOXA9
hsa-mir-373	ATAD2, TMEM100, PBK, MIXL1, ELAVL2, LEFTY2, KPNA2, PFKP, SLC7A11
hsa-mir-143	COL1A1
hsa-mir-183	CCNB1
hsa-mir-216b	MCM4

### Functional Enrichment Analysis and PPI Network Construction

**FIGURE 5:**
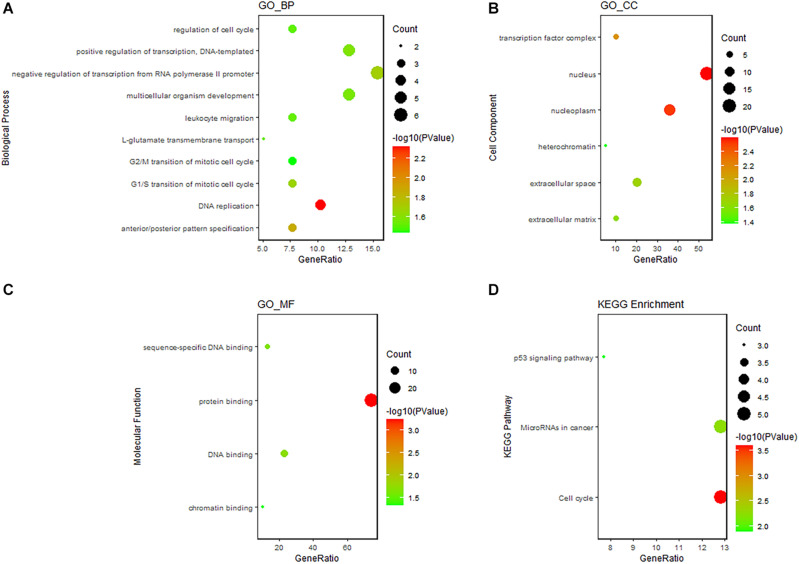
GO and KEGG enrichment analysis of DEmRNAs in the ceRNA network. **(A)** Bubble Plot of BP. **(B)** Bubble Plot of CC. **(C)** Bubble Plot of MF. **(D)** Bubble Plot of KEGG. GO, Gene Ontology; BP, biological processes; CC, cell component; MF, molecular function.

**FIGURE 6:**
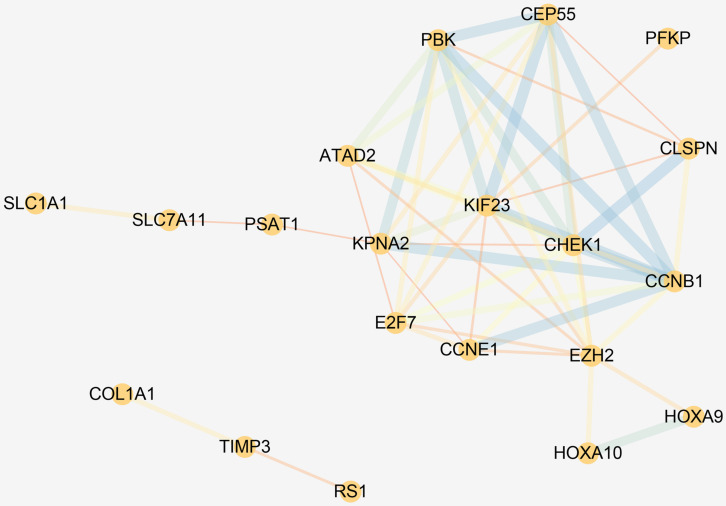
PPI network of the DEmRNAs in the ceRNA network. The circles represent genes, and the lines represent interactions between the proteins encoded by the genes.

Gene Ontology and Kyoto Encyclopedia of Genes and Genomes pathway enrichment analyses revealed that the DEmRNAs involved in the ceRNA network were remarkably associated with 14 BPs, including DNA replication, cell cycle regulation, leukocyte migration, and G2 DNA damage checkpoint. The enrichment of MFs is mainly related to protein binding. We found that the most enriched CC was the nucleus. ceRNA network–related genes were significantly enriched in three KEGG pathways, namely, the p53 signaling pathway, miRNAs in cancer, and cell cycle. The results of functional enrichment analysis are listed in Supplementary Table 3 and shown in Figure 5. Enrichment analysis suggested that the LUAD-specific ceRNA network might be involved in the tumor process by regulating these biological processes and pathways. In addition, to identify interactions of proteins translated from the mRNAs in the ceRNA network, The PPI network was constructed (Figure 6). We found that some genes with high combined score, including CHEK1, CDC25A, CCNE1, CCNB1, and MCM4 were mainly enriched in the “cell cycle” pathway.

### Survival Analysis of DERNAs Involved in the ceRNA Network

**FIGURE 7:**
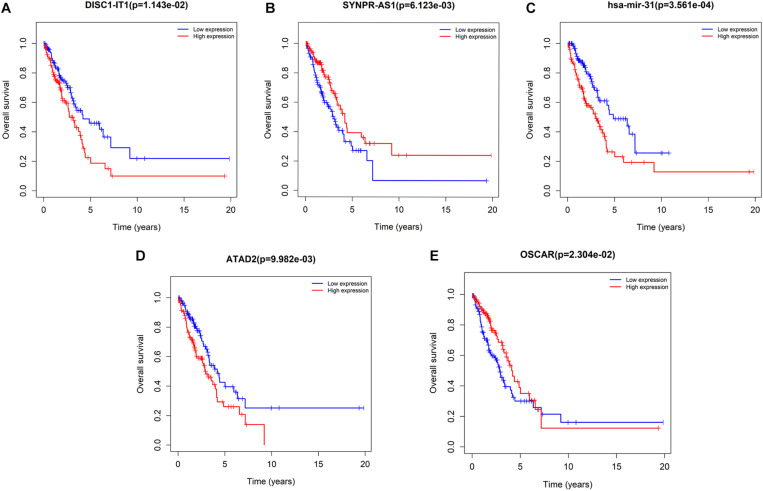
Kaplan–Meier curve analysis of DERNAs for the overall survival in LUAD patients. Five representative DERNAs, including DElnRNAs: **(A)** DISC1-IT1 **(B)** SYNPR-AS1; DEmiRNAs: **(C)** hsa-mir-31; and DEmRNAs: **(D)** ATAD2 **(E)** OSCAR (*p* < 0.05). Horizontal axis: overall survival time, years; vertical axis: overall survival rate.

Univariate survival analysis was performed to identify ceRNA-based biomarkers for LUAD prognosis. As a result, seven DElncRNAs (DISC1-IT1, SYNPR-AS1, H19, LINC00460, LINC00518, DSCR10, and STEAP2-AS1), one DEmiRNA (hsa-mir-31), and 16 DEmRNAs (ATAD2, OSCAR, KIF23, E2F7, PFKP, MCM4, CEP55, CBX2, CCNE1, CLSPN, CCNB1, CDC25A, EZH2, CHEK1, SLC7A11, and PBK) were revealed to be associated with LUAD prognosis. Among these biomarkers, SYNPR-AS1 (lncRNA) and OSCAR (mRNA) were considered protective biomarkers. The remaining lncRNAs, miRNAs, and mRNAs were negatively associated with the overall survival of patients with LUAD. The representative results are shown in Figure 7. The survival curves of the remaining DElncRNAs and DEmRNAs are shown in Supplementary Figures 2, 3, respectively.

## Discussion

Given the unsatisfactory survival rate and high mortality of LUAD, identifying specific biomarkers for the diagnosis and treatment of patients with LUAD is an urgent concern. An increasing number of studies have demonstrated that lncRNAs contribute to tumorigenesis and tumor progression through a variety of pathways. The ceRNA hypothesis proposes that lncRNAs, as ceRNAs, can regulate gene expression in LUAD by competing for shared miRNAs (20, 21). For instance, lncRNA TTN-AS1 is overexpressed in LUAD compared with that in paracancerous tissues, and its overexpression can promote the malignant development of LUAD by regulating the miR-142-5p/cyclin-dependent kinase 5 signaling pathway (22). In addition, lncRNA LINC00355 and lncRNA LINC00466 can target CCNE1 and HOXA10 via sponging miR-195 and miR-144, respectively, thereby promoting the progress of LUAD (23, 24). However, the genome-wide screening of the lncRNA-mediated ceRNA network in LUAD remains lacking.

In this study, we used LUAD expression data downloaded from the TCGA database to identify DElncRNAs, DEmiRNAs, and DEmRNAs between LUAD and corresponding paracancerous tissues. By integrating the interaction between DEmiRNAs and DEmRNAs or DElncRNAs, we constructed a LUAD-specific ceRNA network, which included 157 nodes and 378 edges. The results of GO enrichment analysis revealed that ceRNA-related RNAs were mainly involved in cancer-related biological processes, such as DNA replication, cell cycle regulation, and leukocyte migration. In addition, the DEmRNAs involved in the ceRNA network were significantly enriched in three KEGG pathways, namely, the cell cycle, miRNA, and p53 signaling pathways. The enrichment results suggested that the lncRNA–miRNA–mRNA ceRNA network might regulate the biological processes and pathways of LUAD.

Through survival analysis, seven lncRNAs (DISC1-IT1, SYNPR-AS1, H19, LINC00460, LINC00518, DSCR10, and STEAP2-AS1), one miRNA (hsa-mir-31), and 16 mRNAs (ATAD2, OSCAR, KIF23, E2F7, PFKP, MCM4, CEP55, CBX2, CCNE1, CLSPN, CCNB1, CDC25A, EZH2, CHEK1, SLC7A11, and PBK) were identified to be potential biomarkers of the prognosis of patients with LUAD. Among the prognostic DEmRNAs involved in the ceRNA network, the most significant difference was found in the E2F7 index, which was suggested to be overexpressed in LUAD in our study. Recent studies have confirmed our conclusion: E2F7 has been proven to overexpressed in LUAD, and its expression is regulated by SNHG6 through the competitive sponging of miR-26a-5p, which promotes tumor growth and metastasis (25, 26). E2F7 plays an indispensable role in a variety of tumors, such as gallbladder cancer (27), rectal adenocarcinoma (28), and breast cancer (29), indicating that E2F7 may be a potential prognostic marker. The high expression of E2F7 is related to short patient survival time and can promote NSCLC cell proliferation, migration, and invasion (30). DEmRNAs, including ATAD2, KIF23, MCM4, CCNB1, and CCNE1, are highly expressed in LUAD than in corresponding non-tumor tissues. These highly expressed DEmRNAs have been validated to promote cancer cell proliferation (23, 31–34). Our results indicated that these DEmRNAs can be regulated by the same lncRNA, namely, TCL6, by targeting mir-372, mir-195, mir-373, mir-183, and mir-216b. PFKP and SLC7A11 have been reported to regulate the metabolic levels of lung cancer cells (35, 36). The upregulated expression of CLSPN and CDC25A is related to radioresistance in lung cancer (37, 38). CHEK1 exerts an inhibitory effect on the chemotherapy sensitivity of LUAD cells to cisplatin (39). The high expression levels of PBK, CEP55, and EZH2 have been found to be associated with poor prognosis in LUAD (40, 41). H19 was the most significant prognostic DElncRNA in the ceRNA regulatory network in LUAD. Gao et al. (42) and Liu et al. (43) revealed that H19 promotes the viability and epithelial–mesenchymal transition of LUAD cells. H19 has also been shown to be a prognostic biomarker in bladder cancer (25). LINC00518 and LINC00460 act as oncogenes to facilitate tumor progression, including prostate cancer (44), gastric cancer (45), colon cancer (46), and lung cancer (47). Relevant studies on the function of DISC1-IT1, SYNPR-AS1 DSCR10, STEAP2-AS1, and hsa-mir-31 in cancer do not exist in the literature.

Although we constructed an LUAD-specific ceRNA regulatory network and screened for potential prognostic biomarkers, several limitations remain. First, this study was based merely on the gene expression data downloaded from the TCGA database. Prospective research involving different populations and centers with large patient sizes are needed to validate our results. Second, our research did not involve the clinical characteristics, such as TNM stage and gene mutation, of patients with LUAD. Third, further experimental research is needed to verify the potential biological mechanisms of these ceRNAs in LUAD in the future.

## Conclusion

An LUAD-specific lncRNA-related ceRNA regulatory network was constructed by using bioinformatics methods. We also identified potential prognostic biomarkers, which provided novel insights into the tumorigenesis mechanism of LUAD. Further experimental studies are needed to validate this underlying biological regulatory mechanism in the future.

## Data Availability Statement

The datasets presented in this study can be found in online repositories. The names of the repository/repositories and accession number(s) can be found below: https://portal.gdc.cancer.gov/, The Cancer Genome Atlas; http://www.mircode.org/, The miRcode database; http://mirtarbase.mbc.nctu.edu.tw/, The miRTarBase database; http://www.mirdb.org/, The miRDB database; http://www.targetscan.org/, The TargetScan database; and https://string-db.org/, The STRING.

## Author Contributions

XW and ZS: design and initiation of the study, quality control of data, data analysis and interpretation, and manuscript preparation and editing. HZ and YW: data acquisition. ZY: study concept and design and initiation of the study. All authors: revision and approval of the final version of the manuscript.

## Conflict of Interest

The authors declare that the research was conducted in the absence of any commercial or financial relationships that could be construed as a potential conflict of interest.

## Footnotes

1 https://portal.gdc.cancer.gov/

2 http://www.mircode.org/

3 http://mirtarbase.mbc.nctu.edu.tw/

4 http://www.mirdb.org/

5 http://www.targetscan.org/

## Abbreviations

ceRNA: competing endogenous RNA
DElncRNAs: differentially expressed lncRNAs
DEmiRNAs: differentially expressed miRNAs
DEmRNAs: differentially expressed mRNAs
DERNAs: differentially expressed RNAs
GO: Gene ontology
KEGG: Kyoto Encyclopedia of Genes and Genomes
LUAD: lung adenocarcinoma
MREs: miRNA response elements
ncRNAs: non-coding RNAs
TCGA: The Cancer Genome Atlas.
