# Supplementary material for: Integrated Analysis of lncRNA–Mediated ceRNA Network in Lung Adenocarcinoma
Source: Front Oncol. 2020 Sep 15;10:554759. doi: 10.3389/fonc.2020.554759 (PMC7523091; doi:10.3389/fonc.2020.554759)
Supplement: Supplementary file 1 [file Table_1.DOCX]

**Supplementary Table 1. The Top 10 differentially expressed lncRNAs, miRNAs and mRNAs between tumor and normal tissues in LUAD.**

|  | **Symbol** | **Log2 Fold Change** | **P value** | **Type** |
| --- | --- | --- | --- | --- |
| lncRNAs | LINC00676 | 11.10178151 | 3.75E-06 | Up |
|  | DSCAM-AS1 | 10.07379776 | 3.26E-06 | Up |
|  | LINC01419 | 8.945360435 | 4.03E-05 | Up |
|  | LINC01194 | 8.36192312 | 3.17E-07 | Up |
|  | AL513304.1 | 7.870355056 | 0.001079169 | Up |
|  | LINC01833 | 7.79668001 | 3.29E-12 | Up |
|  | AC026785.3 | 7.793186017 | 6.07E-08 | Up |
|  | BCAR4 | 7.550556434 | 3.64E-06 | Up |
|  | AC011298.1 | 7.480882854 | 6.77E-07 | Up |
|  | AL160271.2 | 7.410825372 | 0.000145722 | Up |
| miRNAs | miR-372 | 8.165867904 | 0.00016018 | Up |
|  | miR-371a | 7.354085259 | 0.005490697 | Up |
|  | miR-105-2 | 7.084407454 | 1.59E-05 | Up |
|  | miR-122 | 7.083956441 | 0.002172957 | Up |
|  | miR-373 | 6.768224267 | 0.001547373 | Up |
|  | miR-105-1 | 6.545944717 | 7.74E-05 | Up |
|  | miR-4652 | 6.22406006 | 9.44E-08 | Up |
|  | miR-9-1 | 6.160479681 | 3.34E-13 | Up |
|  | miR-9-2 | 6.151261161 | 3.18E-13 | Up |
|  | miR-9-3 | 6.148367743 | 3.58E-13 | Up |
| mRNAs | REG4 | 11.318535 | 1.46E-07 | Up |
|  | TFF2 | 10.68178055 | 1.91E-06 | Up |
|  | MAGEA4 | 10.46098165 | 3.41E-05 | Up |
|  | CGA | 10.23765546 | 8.86E-07 | Up |
|  | DEFA5 | 10.02633723 | 0.000356074 | Up |
|  | TRIM48 | 9.90411384 | 0.000112933 | Up |
|  | MAGEA6 | 9.75300025 | 7.55E-07 | Up |
|  | ALB | 9.666699335 | 1.45E-07 | Up |
|  | MAGEA3 | 9.506740871 | 2.65E-07 | Up |
|  | PAGE2 | 9.489083213 | 1.06E-05 | Up |
