# Supplementary material for: Integrated Analysis of lncRNA–Mediated ceRNA Network in Lung Adenocarcinoma
Source: Front Oncol. 2020 Sep 15;10:554759. doi: 10.3389/fonc.2020.554759 (PMC7523091; doi:10.3389/fonc.2020.554759)
Supplement: Supplementary file 2 [file Table_2.DOCX]

**Supplementary Table 2. All the 105 DElncRNAs in the ceRNA network**

| **DElncRNAs** | **logFC** | **P Value** | **FDR** |
| --- | --- | --- | --- |
| ADAMTS9-AS1 | -3.08409 | 5.22E-46 | 2.54E-43 |
| LINC00163 | -3.87092 | 3.58E-40 | 1.21E-37 |
| NAV2-AS2 | -3.17035 | 7.34E-27 | 9.88E-25 |
| LINC00472 | -2.34311 | 2.62E-23 | 2.58E-21 |
| LINC00092 | -2.26402 | 3.49E-23 | 3.28E-21 |
| C5orf64 | -2.33613 | 7.83E-22 | 6.53E-20 |
| LINC00211 | -2.58267 | 2.48E-19 | 1.75E-17 |
| AC020907.1 | 6.109756 | 9.95E-18 | 5.76E-16 |
| PVT1 | 2.489072 | 2.58E-17 | 1.39E-15 |
| AGAP11 | -2.13595 | 6.76E-17 | 3.44E-15 |
| C1orf220 | 2.280105 | 2.13E-16 | 1.04E-14 |
| UBE2Q1-AS1 | 2.118183 | 8.28E-12 | 2.00E-10 |
| C1orf147 | 2.017332 | 6.00E-11 | 1.27E-09 |
| CLDN10-AS1 | 6.337799 | 7.56E-11 | 1.57E-09 |
| LINC00337 | 3.312684 | 1.26E-10 | 2.48E-09 |
| RMST | -2.47086 | 7.63E-10 | 1.30E-08 |
| LINC00525 | 3.611953 | 1.23E-09 | 2.04E-08 |
| AC087269.1 | 3.611953 | 1.29E-09 | 2.12E-08 |
| AC105206.1 | -2.64906 | 2.01E-09 | 3.18E-08 |
| LINC00461 | 3.826666 | 3.41E-09 | 5.08E-08 |
| AC025431.1 | -2.46951 | 1.70E-08 | 2.21E-07 |
| LINC00160 | 3.463884 | 2.31E-08 | 2.94E-07 |
| STEAP2-AS1 | 3.292617 | 2.84E-08 | 3.53E-07 |
| MIR137HG | 6.042659 | 8.33E-08 | 9.30E-07 |
| UCA1 | 5.432528 | 9.64E-08 | 1.05E-06 |
| LINC00501 | 5.646083 | 1.04E-07 | 1.13E-06 |
| FNDC1-IT1 | 3.853057 | 1.49E-07 | 1.54E-06 |
| LINC00460 | 4.771485 | 1.51E-07 | 1.55E-06 |
| HOTAIR | 6.777891 | 2.54E-07 | 2.49E-06 |
| DLX6-AS1 | 5.114279 | 3.47E-07 | 3.28E-06 |
| C20orf197 | 2.949087 | 4.51E-07 | 4.12E-06 |
| ERVH48-1 | 5.246445 | 9.74E-07 | 8.20E-06 |
| C10orf91 | 2.13317 | 1.18E-06 | 9.69E-06 |
| AC104472.1 | 2.269639 | 1.29E-06 | 1.04E-05 |
| LINC00518 | 4.993772 | 2.22E-06 | 1.70E-05 |
| LINC00466 | 4.211257 | 2.53E-06 | 1.90E-05 |
| DSCAM-AS1 | 10.0738 | 3.26E-06 | 2.38E-05 |
| MIAT | 2.196284 | 4.15E-06 | 2.93E-05 |
| LINC00519 | 2.525809 | 4.43E-06 | 3.10E-05 |
| LINC00491 | 5.347523 | 5.96E-06 | 4.02E-05 |
| TMEM78 | 3.019079 | 8.63E-06 | 5.52E-05 |
| LINC00536 | 3.730754 | 9.21E-06 | 5.86E-05 |
| TCL6 | 2.951336 | 1.33E-05 | 8.01E-05 |
| AP002478.1 | 3.837964 | 1.51E-05 | 8.93E-05 |
| EGOT | 2.288529 | 1.73E-05 | 0.0001 |
| LNX1-AS2 | 2.154494 | 2.05E-05 | 0.000116 |
| AC112721.1 | 2.586631 | 2.73E-05 | 0.000149 |
| POU6F2-AS1 | 4.124197 | 3.52E-05 | 0.000185 |
| AC022148.1 | 3.285273 | 3.59E-05 | 0.000188 |
| ABCA9-AS1 | 3.677089 | 4.18E-05 | 0.000214 |
| C21orf91-OT1 | 3.125013 | 9.09E-05 | 0.00042 |
| LINC00184 | 2.147781 | 0.000143 | 0.000621 |
| LINC00355 | 4.625738 | 0.000151 | 0.000649 |
| LINC00524 | 3.386242 | 0.000152 | 0.000652 |
| GRM5-AS1 | 2.747966 | 0.000154 | 0.000657 |
| FLRT1 | 2.250935 | 0.000226 | 0.000914 |
| HNF1A-AS1 | 2.625129 | 0.000278 | 0.001087 |
| KIF25-AS1 | 3.162999 | 0.000315 | 0.001209 |
| LINC00330 | 2.129097 | 0.000351 | 0.001325 |
| TBL1XR1-AS1 | 2.525875 | 0.00036 | 0.001356 |
| DSCR4 | 5.946415 | 0.000438 | 0.001605 |
| LINC00221 | 5.014443 | 0.000514 | 0.001828 |
| LINC00470 | 3.290874 | 0.000577 | 0.002011 |
| AL117190.1 | 2.450451 | 0.000588 | 0.002043 |
| LINC00319 | 2.729626 | 0.000635 | 0.002185 |
| ERVMER61-1 | 5.561449 | 0.000722 | 0.00244 |
| AL163952.1 | 2.018276 | 0.000848 | 0.002798 |
| C11orf44 | 2.477362 | 0.001162 | 0.003656 |
| AC006372.1 | 3.888598 | 0.001416 | 0.004323 |
| LINC00440 | 3.297431 | 0.001805 | 0.005315 |
| LINC00485 | 3.51682 | 0.001917 | 0.005599 |
| AL391152.1 | 2.072924 | 0.002372 | 0.006707 |
| FOXP1-IT1 | 2.327207 | 0.002997 | 0.008148 |
| VCAN-AS1 | 2.167851352 | 0.003367573 | 0.008959382 |
| IL20RB-AS1 | 2.478743 | 0.00343 | 0.009088 |
| CLLU1 | 2.041169 | 0.003438 | 0.009105 |
| DSCR10 | 3.352831 | 0.003674 | 0.009604 |
| HOTTIP | 3.33121 | 0.005944 | 0.014392 |
| C5orf17 | 2.529574 | 0.007333 | 0.017281 |
| DPYD-AS2 | 2.975609 | 0.008393 | 0.019442 |
| ST7-AS2 | 2.205405 | 0.008579 | 0.019807 |
| DISC1-IT1 | 2.008684 | 0.008882 | 0.02041 |
| PHEX-AS1 | 2.318284 | 0.01466 | 0.031258 |
| LINC00200 | 3.74721 | 0.014877 | 0.031661 |
| AL139002.1 | 3.805206 | 0.014918 | 0.031732 |
| AC002511.1 | 2.019699 | 0.016108 | 0.033788 |
| AC061975.6 | 6.767363065 | 3.02E-08 | 3.72E-07 |
| LINC00392 | 5.988733786 | 0.0012881 | 0.003982599 |
| NAALADL2-AS2 | 5.211222015 | 9.60E-07 | 8.10E-06 |
| AL513123.1 | 5.137153728 | 3.28E-06 | 2.38E-05 |
| LINC00393 | 4.530403389 | 0.001281935 | 0.003967742 |
| AC084262.1 | 4.193329385 | 7.23E-08 | 8.22E-07 |
| MYCNOS | 3.621440366 | 6.29E-05 | 0.000306781 |
| LSAMP-AS1 | 3.399560692 | 0.000339945 | 0.001288436 |
| AL354707.1 | 3.387766255 | 1.75E-12 | 4.92E-11 |
| AC100791.1 | 2.880656897 | 0.000390831 | 0.001460695 |
| H19 | 2.691459097 | 0.001332299 | 0.004107649 |
| AC012640.1 | 2.534030962 | 3.89E-08 | 4.67E-07 |
| AC012050.1 | 2.533664128 | 0.020717006 | 0.041763231 |
| CHODL-AS1 | 2.413280373 | 0.022128662 | 0.04419169 |
| SYNPR-AS1 | 2.060010669 | 3.25E-05 | 0.000173399 |
| MED4-AS1 | -2.552278129 | 1.12E-36 | 2.97E-34 |
| E2F3-IT1 | 2.47627 | 0.016167 | 0.033876 |
| LINC00488 | 3.266505 | 0.016371 | 0.033876 |
| AC004832.1 | -2.885669433 | 4.12E-18 | 2.51E-16 |
