# Supplementary material for: Integrated Analysis of lncRNA–Mediated ceRNA Network in Lung Adenocarcinoma
Source: Front Oncol. 2020 Sep 15;10:554759. doi: 10.3389/fonc.2020.554759 (PMC7523091; doi:10.3389/fonc.2020.554759)
Supplement: Supplementary file 3 [file Table_3.docx]

**Supplementary Table 3. GO and KEGG pathway analyses of DEmRNAs in the ceRNA network**

| **Category** | **ID** | **Description** | **Count** | **P value** |
| --- | --- | --- | --- | --- |
| **Biological process** | GO:0006260 | DNA replication | 4 | 0.004762 |
|  | GO:0009952 | anterior/posterior pattern specification | 3 | 0.013401 |
|  | GO:0000122 | negative regulation of transcription from RNA polymerase II promoter | 6 | 0.020003 |
|  | GO:0000082 | G1/S transition of mitotic cell cycle | 3 | 0.021194 |
|  | GO:0045893 | positive regulation of transcription, DNA-templated | 5 | 0.025969 |
|  | GO:0007275 | multicellular organism development | 5 | 0.026952 |
|  | GO:0089711 | L-glutamate transmembrane transport | 2 | 0.028279 |
|  | GO:0050900 | leukocyte migration | 3 | 0.029549 |
|  | GO:0060065 | uterus development | 2 | 0.030422 |
|  | GO:0051726 | regulation of cell cycle | 3 | 0.030446 |
|  | GO:0060216 | definitive hemopoiesis | 2 | 0.034693 |
|  | GO:0000086 | G2/M transition of mitotic cell cycle | 3 | 0.036538 |
|  | GO:0031572 | G2 DNA damage checkpoint | 2 | 0.043182 |
|  | GO:0060045 | positive regulation of cardiac muscle cell proliferation | 2 | 0.047399 |
| **Cellular component** | GO:0000792 | heterochromatin | 2 | 0.04291 |
|  | GO:0005667 | transcription factor complex | 4 | 0.007513 |
|  | GO:0005654 | nucleoplasm | 14 | 0.003032 |
|  | GO:0005634 | nucleus | 21 | 0.002562 |
|  | GO:0005615 | extracellular space | 8 | 0.019796 |
|  | GO:0031012 | extracellular matrix | 4 | 0.02354 |
| **Molecular function** | GO:0005515 | protein binding | 29 | 5.89E-04 |
|  | GO:0003677 | DNA binding | 9 | 0.018977 |
|  | GO:0003682 | chromatin binding | 4 | 0.046742 |
|  | GO:0043565 | sequence-specific DNA binding | 5 | 0.021629 |
| **KEGG pathways** | hsa05206 | MicroRNAs in cancer | 5 | 0.00564 |
|  | hsa04115 | p53 signaling pathway | 3 | 0.012931 |
|  | hsa04110 | Cell cycle | 5 | 2.53E-04 |
