# Supplementary material for: Integrated Analysis of lncRNA–Mediated ceRNA Network in Lung Adenocarcinoma
Source: Front Oncol. 2020 Sep 15;10:554759. doi: 10.3389/fonc.2020.554759 (PMC7523091; doi:10.3389/fonc.2020.554759)
Supplement: Supplementary file 4 [file Data_Sheet_1.docx]

**
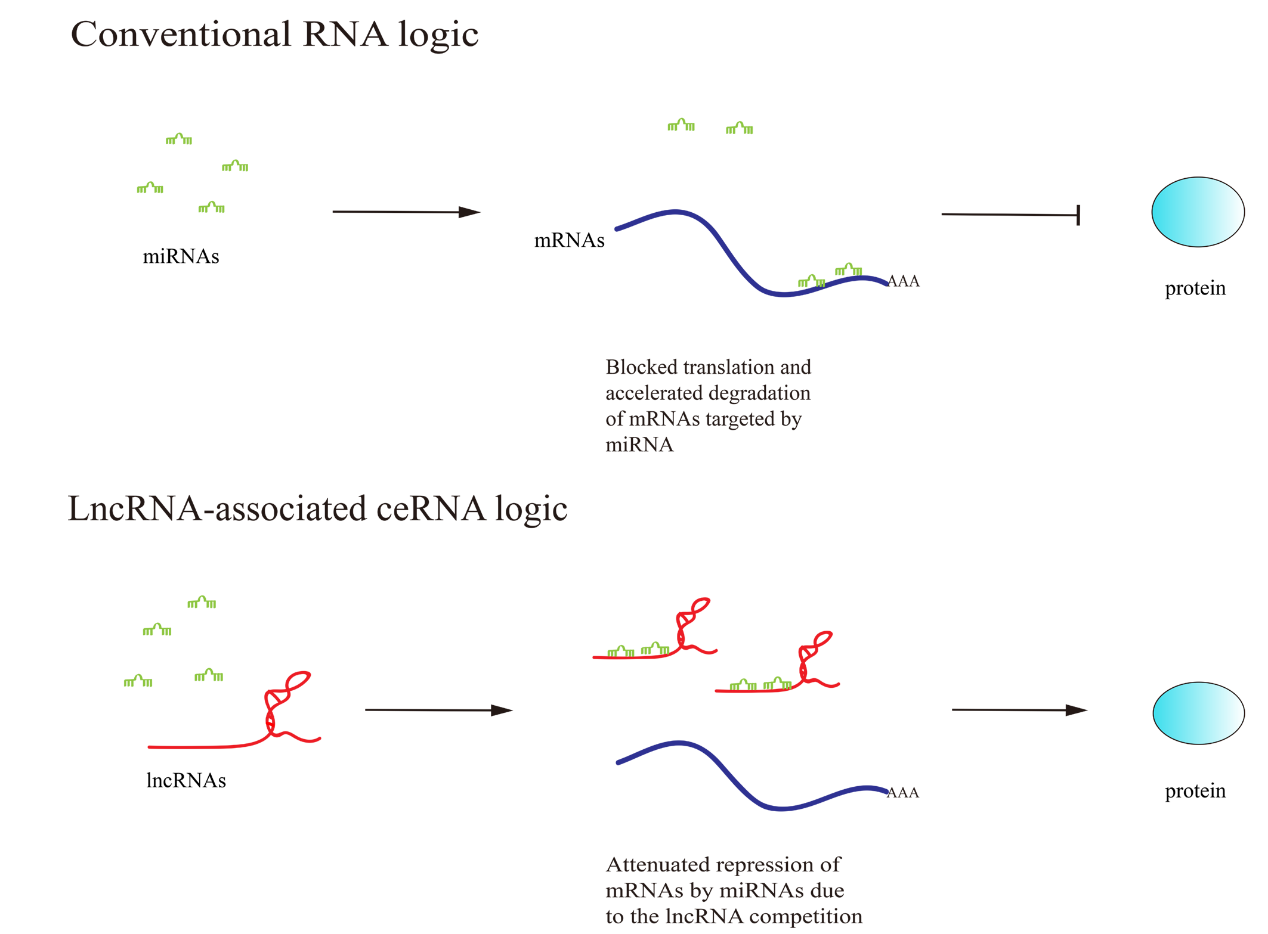
**

**Supplementary Figure 1.** The schematic diagram of lncRNA-mediated ceRNA regulatory network


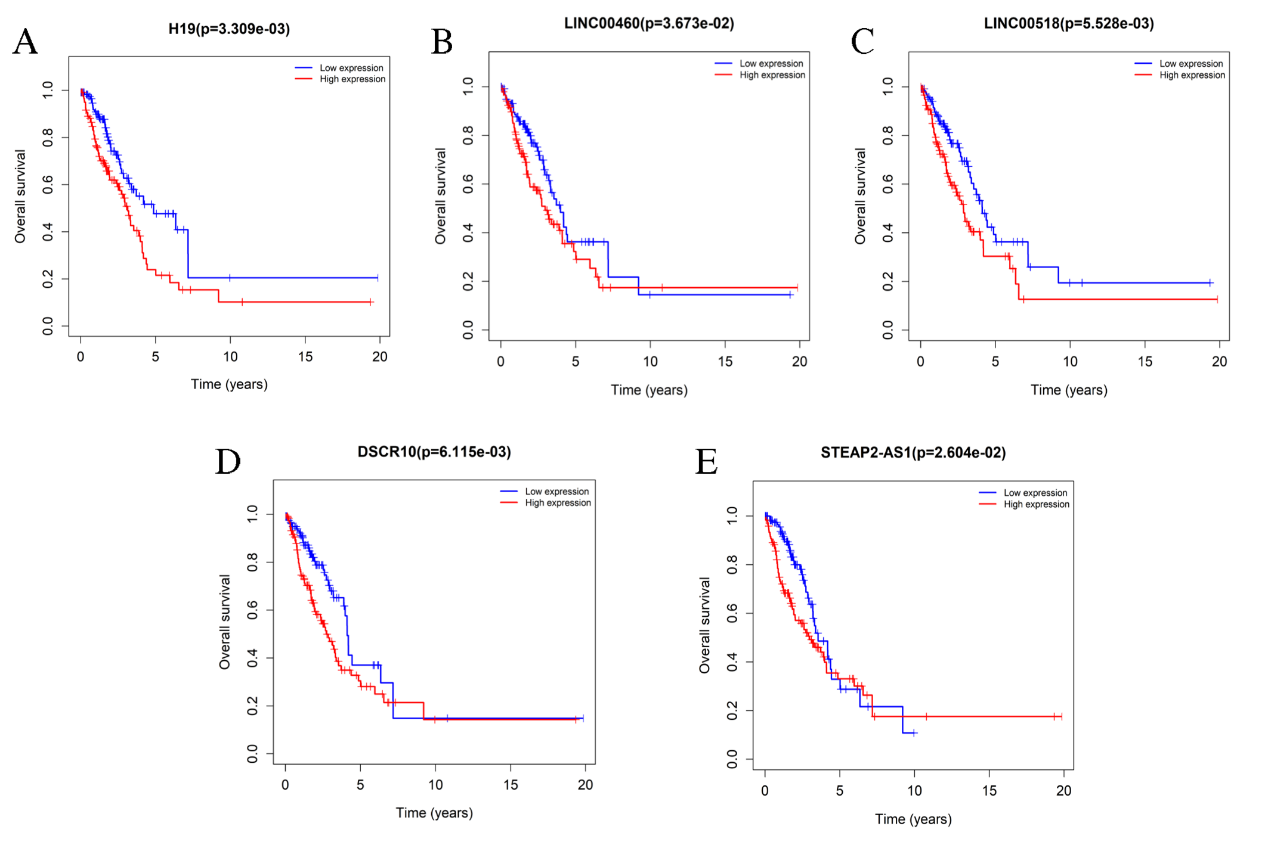


**Supplementary Figure 2.** Kaplan-Meier curve analysis DElncRNAs for the overall survival in LUAD patients. **(A)** H19 **(B)** LINC00460 **(C)** LINC00518 **(D)** DSCR10 **(E)** STEAP2-AS1 were showed (p<0.05). Horizontal axis: overall survival time, years; Vertical axis: overall survival rate.


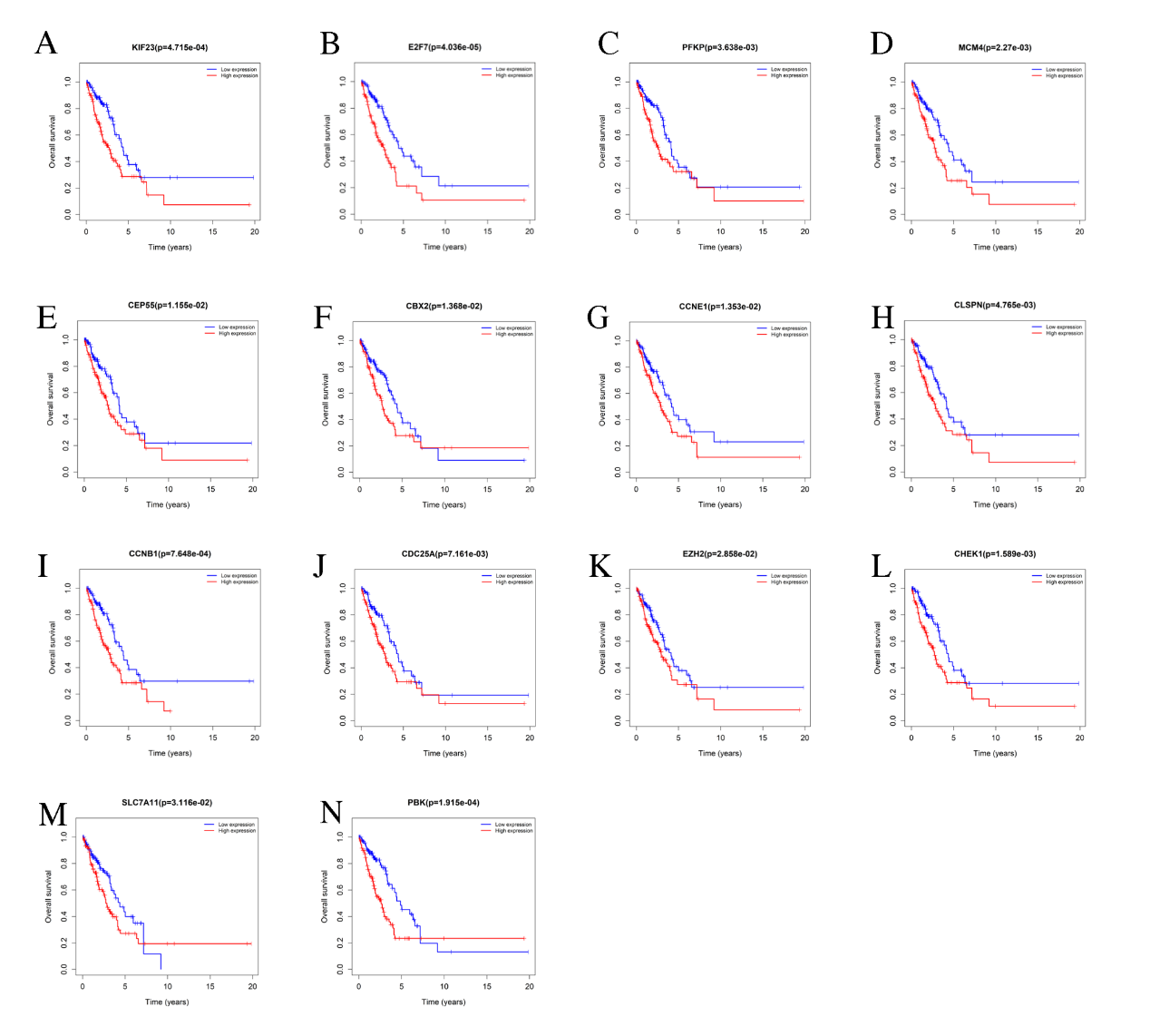


**Supplementary Figure 3.** Kaplan-Meier curve analysis of DEmRNAs for the overall survival in LUAD patients. including **(A)** KIF23 **(B)** E2F7 **(C)** PFKP **(D)** MCM4 **(E)** CEP55 **(F)** CBX2 **(G)** CCNE1 **(H)** CLSPN **(I)** CCNB1 **(J)** CDC25A **(K)** EZH2 **(L)** CHEK1 **(M)** SLC7A11 **(N)** PBK (p<0.05). Horizontal axis: overall survival time, years; Vertical axis: overall survival rate.
